# Self-assembled oxide films with tailored nanoscale ionic and electronic channels for controlled resistive switching

**DOI:** 10.1038/ncomms12373

**Published:** 2016-08-05

**Authors:** Seungho Cho, Chao Yun, Stefan Tappertzhofen, Ahmed Kursumovic, Shinbuhm Lee, Ping Lu, Quanxi Jia, Meng Fan, Jie Jian, Haiyan Wang, Stephan Hofmann, Judith L. MacManus-Driscoll

**Affiliations:** 1 Department of Materials Science and Metallurgy, University of Cambridge, 27 Charles Babbage Road, Cambridge CB3 0FS, UK; 2 Department of Engineering, University of Cambridge, 9 J.J. Thomson Avenue, Cambridge CB3 0FA, UK; 3 Sandia National Laboratory, Albuquerque, New Mexico 87185, USA; 4 Center for Integrated Nanotechnologies, Los Alamos National Laboratory, Los Alamos, New Mexico 87545, USA; 5 Department of Electrical and Computer Engineering, Texas A&M University, College Station, Texas 77843, USA; 6 School of Materials Engineering, Purdue University, West Lafayette, Indiana 47907, USA

## Abstract

Resistive switches are non-volatile memory cells based on nano-ionic redox processes that offer energy efficient device architectures and open pathways to neuromorphics and cognitive computing. However, channel formation typically requires an irreversible, not well controlled electroforming process, giving difficulty to independently control ionic and electronic properties. The device performance is also limited by the incomplete understanding of the underlying mechanisms. Here, we report a novel memristive model material system based on self-assembled Sm-doped CeO_2_ and SrTiO_3_ films that allow the separate tailoring of nanoscale ionic and electronic channels at high density (∼10^12^ inch^−2^). We systematically show that these devices allow precise engineering of the resistance states, thus enabling large on–off ratios and high reproducibility. The tunable structure presents an ideal platform to explore ionic and electronic mechanisms and we expect a wide potential impact also on other nascent technologies, ranging from ionic gating to micro-solid oxide fuel cells and neuromorphics.

## Abstract (web summary)

Metal oxide resistive switches rely on the migration of oxygen vacancies and electrons under applied voltage. Here, Cho *et al*. use nanocomposites to control the electronic and ionic conductivities in spatially distinct channels, and fabricate memristors with high on/off ratios and reproducibility.

## Graphical abstract

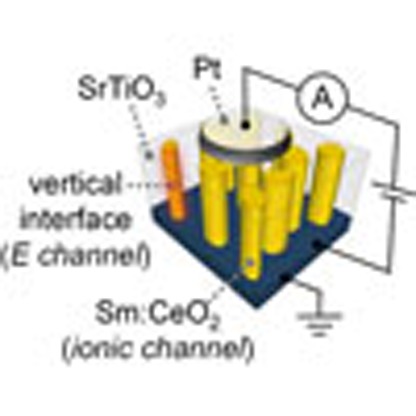

A range of emerging non-volatile random-access memory technologies for faster, higher density and low energy memory are now being explored worldwide to overcome the emerging challenges of scale, speed and energy of today's flash floating-gate metal-oxide-semiconductor field-effect transistor technology based on silicon. Among them, resistive random access memory (ReRAM, also termed as memristive device) is one of the promising candidates for the future non-volatile random-access memory. ReRAM has the potential for high-density integration, fast operation, low-power consumption, and fabrication compatibility with silicon complementary metal-oxide-semiconductor technology^1^. In addition, by mimicking biological neurons, memristive devices have the potential to combine logic+memory operations and hence are of interest in future cognitive computing^2^.

Metal oxide resistive switching structures have been widely studied for ReRAM. They have several advantages over phase change memories, ferroelectric RAMs and magnetoresistive RAMs (ref. 3), but still there is a lack of reproducibility in their properties. They also typically exhibit lower endurance than the aforementioned alternatives and need a high-voltage (5–20 V) electroforming process^4^,^5^. The understanding of memristive switching in oxide materials is also incomplete. Various models have been proposed such as filamentary conduction, charge trapping defects states, trap-controlled space-charge-limited current and a change of a Schottky-like barrier^3^,^6^. In fact, these effects are not mutually exclusive, the essential feature of all of the aforementioned models being the migration of oxygen vacancies and electrons under an applied electric field^3^,^7^,^8^,^9^,^10^,^11^.

For valence change resistive switching, the undesired but essential electroforming process which induces random filamentary conduction channels in oxide films induces a redox reaction leading to reduction of the oxygen content in the film to form positively charged oxygen vacancies plus neutralizing electronic carriers^5^,^8^,^12^,^13^,^14^. However, the coupling of the ions and electrons makes the understanding of the resistive switching mechanism unclear. Also, it is difficult to independently control ionic and electronic properties of such channels and the stochastic nature of the electroforming process makes the understanding of the switching process more difficult.

To strongly advance the understanding of resistive switching, we propose a structure in which ionic and electronic channels are decoupled into separate channels and electroforming is inherently not required. Hence, this work is focused on engineering and testing such a structure as a model material system. This was done through creating vertical heteroepitaxial nanocomposite (VHN) films. We show that unique and highly tunable memristive properties result in which the electronic current is controlled by the ionic vacancy concentration in the ionic nanochannels. The structure allows information to be encoded in the confined ionic nanochannels which is closer to nature's purely ionically based information transfer of chemical synapses and hence gives promise for future cognitive computing devices.

50 Sm-doped CeO_2_ (SDC): 50 SrTiO_3_ (STO) (atomic ratios of Sm to Ce: 0, 0.1, 0.2 and 0.3 in SDC) was chosen as the model nanocomposite system to study. In this system, SDC nanocolumns play the ionic role, and the vertical interfaces between SDC and STO play the electronic role in resistive switching. SDC is a well-known oxygen ionic conductor with tunable vacancy concentration depending on the Sm doping level^15^,^16^,^17^. Recently, we discovered high temperature (from 360 to 673 K) ionic conduction in SDC:STO nanocomposites, which grow very easily by self-assembly from a single-pulsed laser deposition target^18^. It was found that very fast conduction SDC ionic nanopillars formed in the STO film matrix. On the other hand, the resistive switching behaviour of the SDC:STO structures was not explored before.

Here, at room temperature, we exploit the high ionic conduction in the SDC nanopillars as well as electronic conduction at the SDC/STO interface, to form, for the first time, a separate ionic and electronic nanochannel device. In these structures, to tune the Schottky barrier we modify the nanopillar ionic conduction by using different Sm doping levels in the SDC. We demonstrate that the SDC:STO structures are electroforming-free with high endurance. Moreover, we demonstrate for the first time that the concentration of mobile oxygen vacancies precisely controls the low-resistance state (LRS). The novel structures have wide application beyond resistive switching to other electronics and energy ionotronic technologies where vertical, fast ionic or tunable mixed (ionic and electronic) conduction is required, for example, solid state ionic gating, fuel cell cathodes and neuromorphic computing^19^.

## Results

### Formation of SDC:STO vertical heteroepitaxial nanocomposites

We first present the results of an optimum film (in terms of resistive switching): 20 at.% SDC:STO VHN film grown on 0.5 wt.% Nb-doped SrTiO_3_ (Nb:STO), using a film growth rate of 0.03 nm s^−1^ by pulsed laser deposition. As we show later, 20% Sm is the optimum doping concentration for the highest ionic conduction (highest mobile vacancy concentration), and also that growth rate is important for controlling crystalline perfection, both of these factors being very important for optimal device performance.

**Figure 1:**
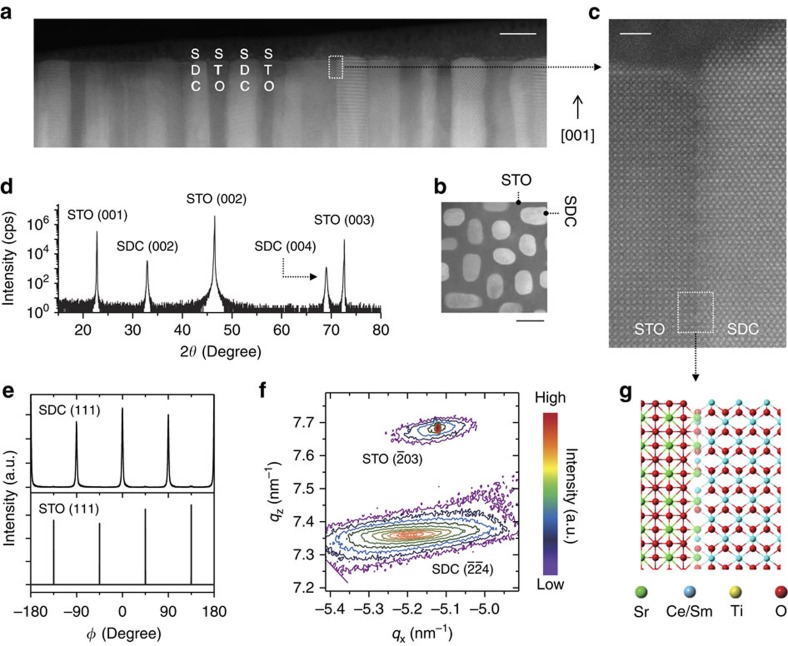
Structural characterizations of VHN. Structural properties of 20 at.% SDC:SrTiO_3_ (STO) VHN from 0.03 nm s^−1^ growth rate which was the optimized film from the point of view of a high on/off ratio on resistive switching. (**a**,**b**) Phase ordering in cross-sectional view and plan view of SDC:STO, as revealed by STEM HAADF images. Scale bars, 30 nm. (**c**) High-resolution HAADF image of the vertical interface. Scale bar, 2 nm. (**d**) XRD *ω*-2*θ* scan of SDC:STO nanocomposite film. (**e**) 360° *ϕ*-scans of the (111) peak of STO and the (111) peak of SDC. (**f**) RSM around the STO(-203) reflection. (**g**) Crystallographic model of a SDC:STO VHN around vertical interface between SDC and STO.

Figure 1a,b show scanning transmission electron microscopy (STEM) high-angle annular dark-field (HAADF) images in a cross-sectional view and plan view, respectively, indicating phase separation and vertically aligned phases (see also Supplementary Fig. 1). The higher atomic number phase, namely the SDC, is brighter than STO. Figure 1c is a highly magnified HAADF image of an SDC/STO interface, showing an atomic-scale sharp and epitaxial interface with an orientational relationship that the SDC [001] axis is parallel to the STO [001] axis and the SDC [100] axis is parallel to the STO [110] axis. The three-dimensional alignment nature of the VHN films was studied using four-circle X-ray diffraction (XRD). The XRD *ω*-2*θ* scan of 20 at.% SDC:STO VHN film with a nominal thickness of ∼200 nm on Nb:STO shows only (00*l*) peaks of SDC and STO (Fig. 1d) without traces of other phases or orientations, confirming their high degree of crystallographic orientation and no intermixed crystalline phases.

The lattice constants of bulk cubic SDC and STO are 5.433 Å in JCPDS # 75-0158 and 3.905 Å in JCPDS # 35-0734, respectively. Thus, the SDC nanopillars are grown on the Nb:STO substrate with an energetically favourable 45° in-plane rotation to minimize in-plane lattice mismatch (1.6%), as shown by the *ϕ*-scan (Fig. 1e). For the SDC:STO film on the Nb:STO further structural information was obtained from reciprocal space maps (RSMs) around STO(03) (Fig. 1f). The SDC (4) peak was observed in the lower *q*_*z*_ region than the (03) peak of the STO. The out-of-plane and the in-plane lattice parameters of SDC were calculated to be 5.430 and 5.443 Å, respectively, from a combination of the *ω*-2*θ* scan and the RSM, corresponding to moderate compression along the out-of-plane direction (0.05%) and moderate expansion along the in-plane direction (0.18%). Figure 1g shows a crystallographic model of the vertical SDC(100)//STO(110) interface based on the STEM and XRD analyses.

### Resistive switching behaviour in nanocomposite-based device

**Figure 2:**
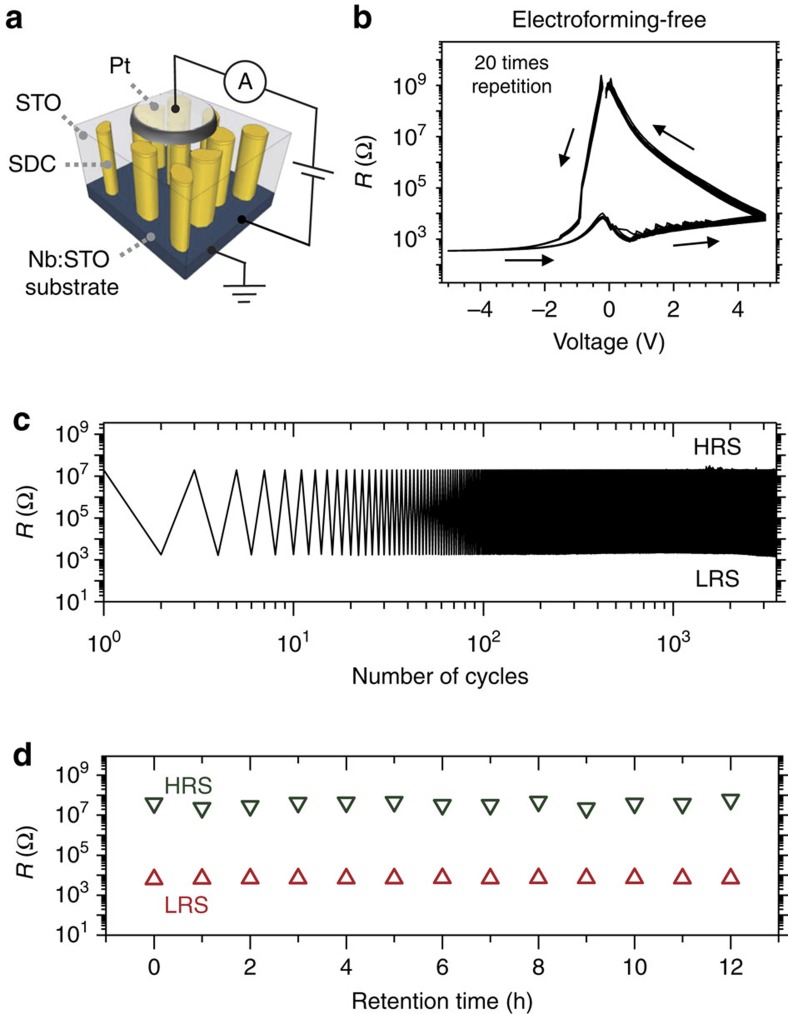
Resistive switching of VHN-based device. (**a**) Schematic of the measurement configuration of SDC:SrTiO_3_ (STO) VHN device. (**b**) Electroforming-free *R*–*V* hysteresis loops in SDC:STO VHN device. (**c**) Uniform resistance variation with repeated electrical cycles. (**d**) The retention characteristics of both resistance states.

Figure 2a,b show a schematic illustration for a (Pt (top)/20 at.% SDC:STO VHN film (nominal thickness: 30 nm)/Nb:STO (bottom)) device and repeated *R*–*V* scans of the device, respectively. For all the electrical measurements, bipolar voltage signals were applied to the circular top Pt electrode with a diameter of 50 μm while the bottom Nb:STO electrodes were grounded. The device exhibited non-volatile resistive switching as a function of voltage (*V*). An electroforming process is not required for reproducible resistive switching properties. One of the curves shown in Fig. 2b was the first scan from the pristine state which was almost the same as the successive *R*–*V* curves. The device was switched to LRS by applying a negative bias and switched to high-resistance state (HRS) by applying a positive bias. This counterclockwise resistance variation has been reported for a number of oxygen vacancy migration-based resistive switching systems^11^,^12^,^13^,^14^. The device exhibited uniform resistance variations for over 10^3^-cycles (Fig. 2c with alternative voltage pulses of –5 and +5 V for switching and a read voltage of –0.3 V), and retention of the original resistance state without degradation (Fig. 2d). In contrast to these devices, plain SDC and STO films required electroforming processes for reversible resistive switching and after electroforming they showed lower on–off ratios and poorer endurance (Supplementary Fig. 2). XRD *ω*-2*θ* scans for an SDC film and an STO film on Nb:STO are provided in Supplementary Fig. 3.

**Figure 3:**
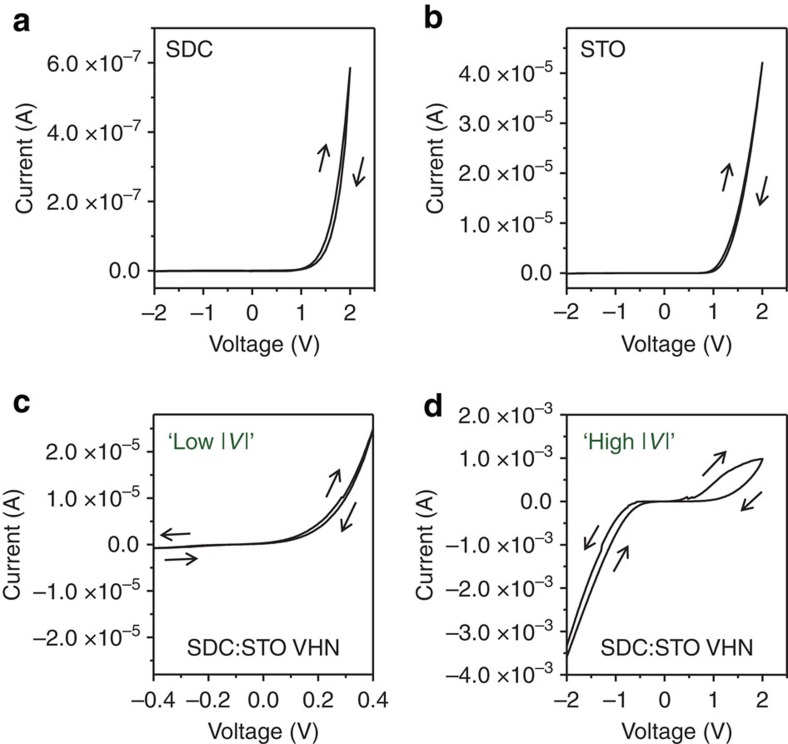
***I*****–*****V*** **curves of devices before electroforming.** (**a**) Plain 20 at.% SDC film device. (**b**) Plain SrTiO_3_ (STO) film device. (**c**,**d**) 20 at.% SDC:STO VHN firm device.

To study conduction and resistive switching mechanisms in the VHN films, we compare optimized VHN films and plain films formed from individual phases at first. Before electroforming, the plain film structures: [Pt/20 at.% SDC film/Nb:STO] and [Pt/STO film/Nb:STO] both showed asymmetric and rectifying *I*–*V* characteristics (Fig. 3a,b, respectively), as expected. The results are consistent with that expected for Schottky-like barriers on top electrode interfaces because SDC and STO are *n*-type materials, Pt is a high work-function metal and the bottom interface with Nb:STO substrate is an ohmic contact^3^,^10^,^11^,^20^.

Turning now to the case of the Pt/SDC:STO VHN/Nb:STO devices, the SDC nanocolumns and the STO matrix are *n*-type semiconductors and their vertical interface regions are also *n*-type oxides due to the high concentration of oxygen vacancies^20^. Thus, by considering only these facts (not taking into account the presence of mobile oxygen vacancies), asymmetric *I*–*V* curves of the VHN structure would be expected to be similar to those observed for the plain SDC and STO devices. Under a small applied bias, an asymmetric and rectifying characteristic was shown (Fig. 3c). The highly asymmetric *I*–*V* curve indicates an interface-limited conduction of the VHN device rather than a bulk-limited conduction. There are four main kinds of interface-limited conduction mechanisms: Schottky emission, direct tunnelling, Fowler-Nordheim tunnelling and interface-limited trap-assisted tunnelling^21^,^22^. The direct tunnelling and Fowler-Nordheim tunnelling can be ruled out in our case because our oxide layers are thick (⩾30 nm). The current density across a Schottky barrier for an applied bias voltage *V*^23^.


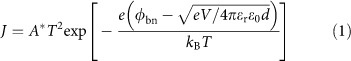


where *A* is the effective Richardson constant, *T* is the absolute temperature, *k*_B_ is Boltzmann's constant, *e* is the electron charge, *eφ*_bn_ is the height of the Schottky barrier, *ɛ*_r_ is the dielectric constant of the film, *ɛ*_0_ is the permittivity of free space and *d* is the sample thickness. If a Schottky barrier controls the current, a semilog plots of *J*/*T*^2^ (or *I*) versus *V*^1/2^ can be fitted by a straight line. The semilog plot of *I* versus *V*^1/2^ for the VHN device indeed shows a straight line fit (Supplementary Fig. 4). In addition, a clear non-linear relationship between log *I* versus *V*^–1^ rules out the trap-assisted tunnelling mechanism^24^. Therefore, in the SDC:STO VHN device, the intrinsic conduction in the HRS is mainly governed by Schottky emission. Interestingly, as the absolute value of negative bias increased, the current dramatically increased, resulting in a completely different *I*–*V* curve shape (Fig. 3d) from the small applied bias case (Fig. 3c). In addition, the *I*–*V* scan with an applied bias range of ±2 V showed a clear hysteresis.

Figure 3 shows that the current in the VHN device was 2–4 orders of magnitude higher than in the plain STO and SDC devices (with the same film thickness), respectively, using the same voltage amplitudes. This indicates that the interface between the Pt and VHN vertical interfaces has a narrower barrier width and/or lower barrier height than those of plain films because electronic conduction in both plain and VHN cases are mainly governed by Schottky emission^20^,^25^. This is supported by conductive atomic force microscopy (c-AFM) measurements indicating higher electronic conductivity along the vertical hetero-interfaces of the VHN film (Supplementary Fig. 5). Electronic conduction of STO can be easily tuned, among other oxides, by chemical doping of a small amount of dopants or by increasing oxygen vacancy concentrations^26^,^27^,^28^,^29^,^30^. The most likely reason for the higher conductivities of the STO:SDC vertical interfaces is a higher concentration of oxygen vacancies along the vertical interfaces by structural mismatch of the two materials (STO: perovskite, *c*=0.3905, nm, SDC: fluorite, *c*=0.5430, nm) than that of the bulk STO part, leading to higher carrier densities along the vertical interfaces^20^ (Supplementary Fig. 6). We note that in the VHN system, ionically conducting^18^ and electronically insulating vertical SDC nanocolumns are embedded in an ionically and electronically insulating, but interfacially electronically conducting, SrTiO_3_ (STO) matrix. Device area-dependent-resistance values were measured (Supplementary Fig. 7). It was found the resistance values in HRS and LRS decreased with increasing Pt electrode area, which is consistent with the fact that our devices have uniformly distributed electronic nanochannels.

To investigate the modulation of the Schottky barrier between the SDC–STO interface and the Pt electrode, we extracted the barrier height, *eφ*_bn_=*k*_B_*T* ln(*AT*^2^/*J*_o_), where *J*_o_ is a saturation current density, from *I*–*V* curves with different temperatures (293, 313, 333 and 353 K)^31^,^32^. For simplicity, we used the effective Richardson constant of *A*=120 A cm^−2^ K^−2^. The increasing absolute value of the negative sweep voltage caused the barrier height (with positive bias) to decrease (Supplementary Fig. 8), which implies that the oxygen vacancy accumulation at the top interface is the cause of the barrier height decrease.

### Separate ionic and electronic nanochannel model

**Figure 4:**
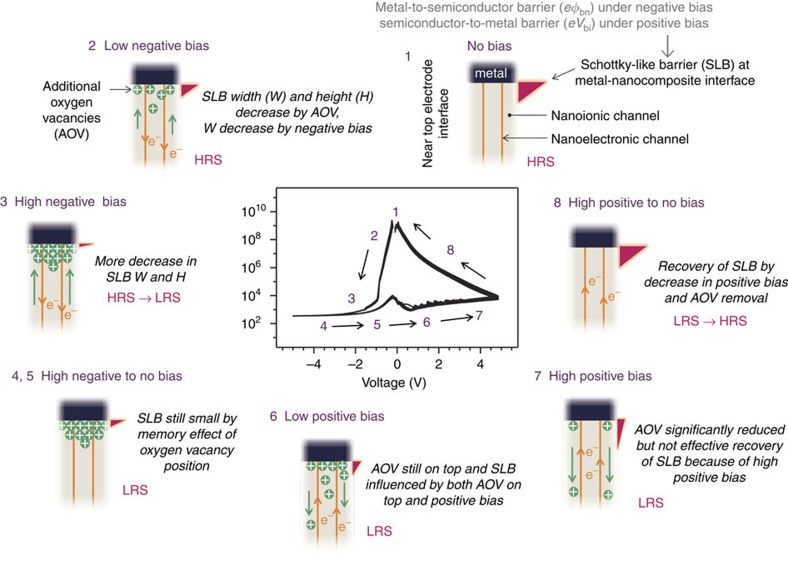
Separate ionic and electronic nanochannel model. Schematic diagram of separate ionic and electronic nanochannel model for resistive switching of the SDC:SrTiO_3_ VHN film device. The simplification of SDC:SrTiO_3_ VHN film device is depicted in Supplementary Fig. 9. HRS, high-resistance state; LRS, low-resistance state.

Based on the aforementioned results, we propose a separate ionic and electronic nanochannel model for the resistive switching mechanism of SDC:STO VHN (SDC: ionic nanochannel, SDC–STO vertical interface: electronic nanochannel)-based devices. Overall, the observed resistive switching behaviour in the SDC:STO VHN device can be explained by the modulation of the interfacial electronic barrier formed on the interface between the top Pt electrode and VHN film due to the migration of oxygen vacancies to or away from the top Pt electrode under the applied electric field. Figure 4 shows separate ionic and electronic nanochannel model for resistive switching of the SDC:STO VHN film device. The simplification of schematic showing cross-sectional SDC:STO VHN film device by discarding bulk STO parts is depicted in Supplementary Fig. 9. The simplification is based on the following features. First, the plain SDC and plain STO showed much lower electronic conductivities than the nanocomposite case and required the electroforming processes for the reversible resistive switching behaviours (Fig. 3 and Supplementary Fig. 2). Second, the electronically conductive channels in the SDC:STO nanocomposite devices are the vertical interfaces of SDC and STO, as shown in Supplementary Fig. 5. The bulk STO and SDC parts are more electronically insulating compared with their vertical interfaces. Third, from the ionic point of view, the ionic conductivities of bulk STO in the SDC:STO vertical nanocomposite films are much lower than those of the SDC parts (SDC columns)^18^.

Assuming an ideal interface, without Fermi pinning, electrons face a metal-to-semiconductor barrier, *eφ*_bn_=*e*(*φ*_m_−χ), where *φ*_m_ is the metal work function and *χ* is the semiconductor electron affinity, under negative bias and a semiconductor-to-metal barrier, *eV*_bi_=*e*(*φ*_m_−*φ*_s_)=*eφ*_bn_−(*E*_C_−*E*_F_), where *φ*_s_, *E*_C_ and *E*_F_ are the work function, the conduction band minimum and the Fermi level of the semiconductor, respectively, under positive bias for electronic conduction. We define these interface electronic barriers as Schottky-like barriers (Fig. 4 and Supplementary Fig. 6). If we assume that there is no mobile dopant (oxygen vacancy) whose position is influenced by the external electric bias and a fully depleted region, in the negative bias condition, *V*_bi_ increases while the barrier width decreases and *φ*_bn_ remains constant. In the positive bias condition, *V*_bi_ decreases while the barrier width increases and *φ*_bn_ still remains constant. In the following we assume that the oxygen vacancy concentration at the Pt/nanoelectronic interface is manipulating the depletion width of a Schottky barrier^33^. When a small negative bias is applied, the width and height of the Schottky-like barrier decreases by oxygen vacancy accumulation at the electrode-nanocomposite interface. The negative bias also decreases the barrier width. As the absolute value of the negative bias increases, the barrier width and height decreases further, causing the device to go into the LRS. Although the absolute value of negative bias decreases, the barrier is still small owing to the memory effect of oxygen vacancy positions because there is no strong driving force for oxygen vacancy migration towards the bottom electrode. When a small positive bias is applied to the device, additional oxygen vacancies are still on top due to the memory effect. Thus, the barrier is influenced by both oxygen vacancies on the top interface and the positive bias, which maintains the LRS of the nanocomposite device. As the absolute value of the positive bias increases, the oxygen vacancy accumulation is significantly reduced. However, the barrier is not effectively recovered because of the high positive bias. The barrier is influenced by both the removal of the accumulated oxygen vacancies and the positive bias, competitively. When the absolute value of positive bias decreases, the barrier is influenced only by a decrease in the positive bias because there is no oxygen vacancy accumulation on the interface between the top Pt electrode and VHN film. Thus, the barrier height is recovered by a decrease in the positive bias, leading to the HRS of the device. Cyclic *I*-*V* scans of self-assembled SDC:STO VHN devices clearly exhibit oxygen vacancy effects as can be seen from the voltage-dependent resistance behaviour (Fig. 4).

**Figure 5:**
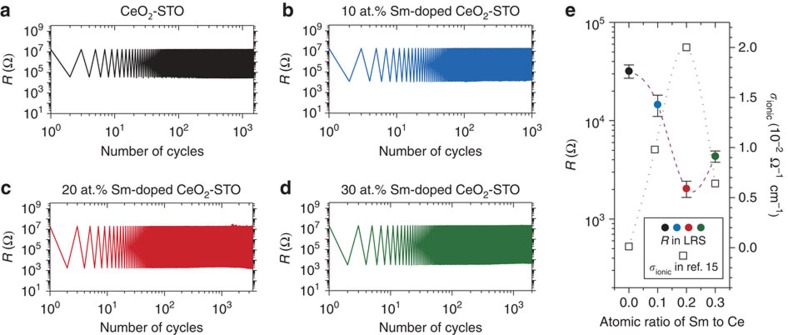
Tuning of resistance values in LRS. Resistance variation with repeated electrical cycles (with applied voltages of −5 and +5 V for switching and a read voltage of −0.3 V) of CeO_2_:SrTiO_3_ VHN film devices with different dopant (Sm^3+^) concentrations in CeO_2_: (**a**) 0 at.% Sm. (**b**) 10 at.% Sm. (**c**) 20 at.% Sm. (**d**) 30 at.% Sm. (**e**) Resistance values in LRS as a function of dopant concentrations (closed circles). Error bars are provided as s.d. We include the ionic conductivity (*σ*_ionic_) values for the films with different dopant concentrations reported in the literature^15^ (open squares).

While the results discussed above underline the importance of oxygen vacancies on resistive switching, to definitively prove and quantify their role, different dopant (Sm^3+^) concentrations in the SDC component (ionic channel) of the films were studied to induce different ionic conductivities (mobile oxygen vacancy concentrations). Figure 5a–d show resistance values of the HRS and the LRS of 0, 10, 20 and 30 at.% SDC:STO VHN films, respectively under alternating pulses of +5 and –5 V. These films all exhibited electroforming-free resistive switching properties. It is observed that the HRS values are very close (to within 15%) for all the compositions studied in this work, as shown in Fig. 5a–d (and Supplementary Fig. 10). Oxygen vacancies are depleted at the interfaces between Pt and VHN films by applying the high positive biases in all the devices. Thus, the resistance values in HRS under high positive biases were similar regardless of intrinsic oxygen vacancy concentrations on the VHN films with the different Sm^3+^ contents. On the other hand, the LRS values decrease when the Sm doping ratios increase from 0 to 20% and then increase again when the doping ratios increase to 30%. Figure 5e shows that there is a direct inverse correlation of LRS with ionic conductivity. The ionic conductivity at 600 °C was used^15^. The values below 600 °C, as is applicable for our measurements, scale with doping concentration in the same order as that of the high temperature data^15^. We note that there will be some Joule heating effects giving very local temperature rises for further enhanced ionic conduction^20^. At 30 at.%, Sm defect association leads to a decrease in mobile oxygen vacancy concentration and hence is consistent with the rise in the resistance value of LRS for this highest doping level^34^. Hence, the role of oxygen vacancy concentration on LRS is indeed clearly proven. Overall, the effect of tuning of the resistance of the LRS while keeping the resistance of the HRS constant is that the on–off ratio is precisely controlled (Supplementary Fig. 10). The maximum on–off ratios achieved are high at ∼10^4^ and are sufficient for practical memory applications^33^.

### Effect of film growth rate on device performance

**Figure 6:**
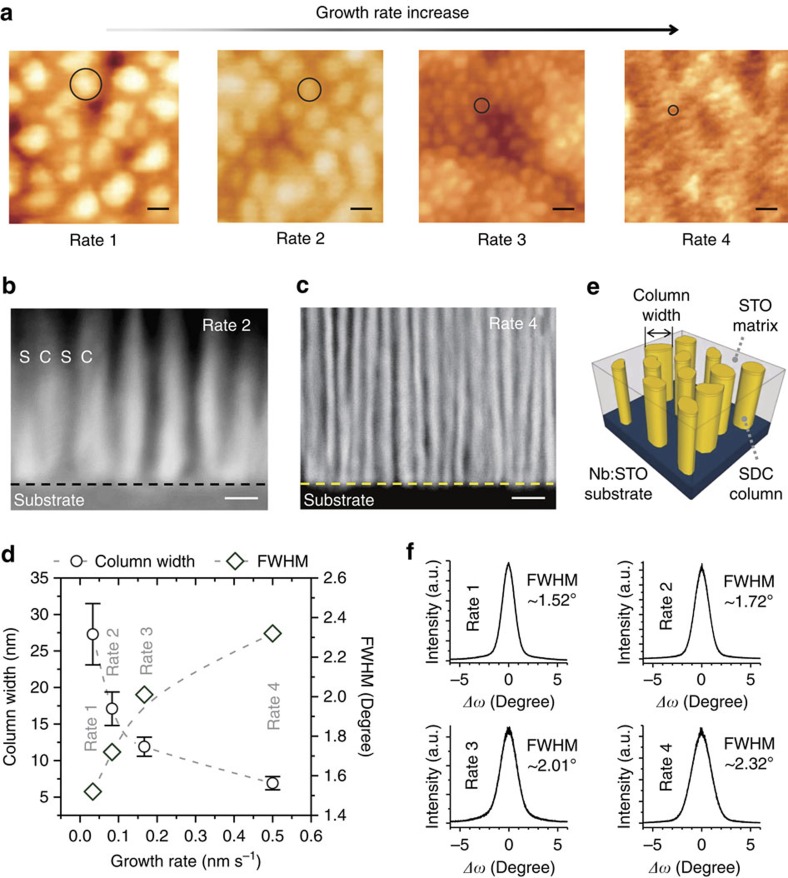
Structural characterizations of VHN deposited from different film growth rates. (**a**) Atomic force microscopy images of 20 at.% SDC:STO VHN films deposited from different film growth rates. Scale bars, 20 nm. (**b**,**c**) Cross-sectional STEM HAADF images of SDC:STO VHN films grown from Rate 2 and Rate 4, respectively. S and C stand for STO and SDC, respectively. Scale bars, 20 nm. (**d**) Column widths and full width at half maximum (FWHM) values in *ω*-rocking curves of SDC:STO VHN films as a function of film growth rates. Error bars are provided as s.d. (**e**) Typical depiction of SDC:STO VHN film on Nb-doped STO substrate, in which the SDC columns are embedded in an STO matrix. (**f**) XRD *ω*-rocking curves of SDC:STO VHN films deposited from different film growth rates.

Finally, the proof of the need for both high ionic conductivity and high quality vertical electronic conducting channels to give forming-free, high endurance and tunable on–off ratio devices is made systematically by deliberately reducing perfection in the structures. This was done by increasing film growth rate from 0.03 to 0.50 nm s^−1^. The growth rates are labelled Rate 1 (for the slowest growth rate) to Rate 4 (for the fastest growth rate). As the growth rates increased, the nanocolumn widths decreased as shown in AFM images (Fig. 6a). TEM cross-section images of the SDC:STO VHNs (Fig. 6b for Rate 2 and 6c for Rate 4) confirmed smaller width columns of SDC in the STO matrices (Fig. 6d,e). In a thin-film growth process, the most thermodynamically stable phases (taking into account epitaxial stabilization) will grow on the substrate during deposition and cooling^35^. There are different phase separation mechanisms; nucleation and growth, spinodal growth and pseudo-spinodal growth depending on the chemical miscibility. In the nucleation and growth mechanism of immiscible two phases, the phase with a higher interfacial energy with the substrate forms nucleation islands that grow into columns, whereas the other phase with a lower interfacial energy undergoes layer-by-layer growth and becomes the plain matrix^36^. A higher growth rate leads to a shorter resting time *τ* of a growth unit and hence a shorter growth unit diffusion length on a substrate, resulting in smaller nucleation islands, and consequently smaller column widths. The diffusion length of a growth unit can be roughly estimated by^37^ 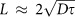, where *D* stands for diffusion coefficient and *τ* for diffusion time. Thus, if the dominant growth mechanism is the diffusion-controlled nucleation and growth, a plot of column width versus (growth rate)^−1/2^ will show straight line fits to the data. Our data agrees well with the diffusion length equation (Supplementary Fig. 11), which is strong evidence of a diffusion-controlled nucleation and growth mechanism of the SDC:STO VHNs.

**Figure 7:**
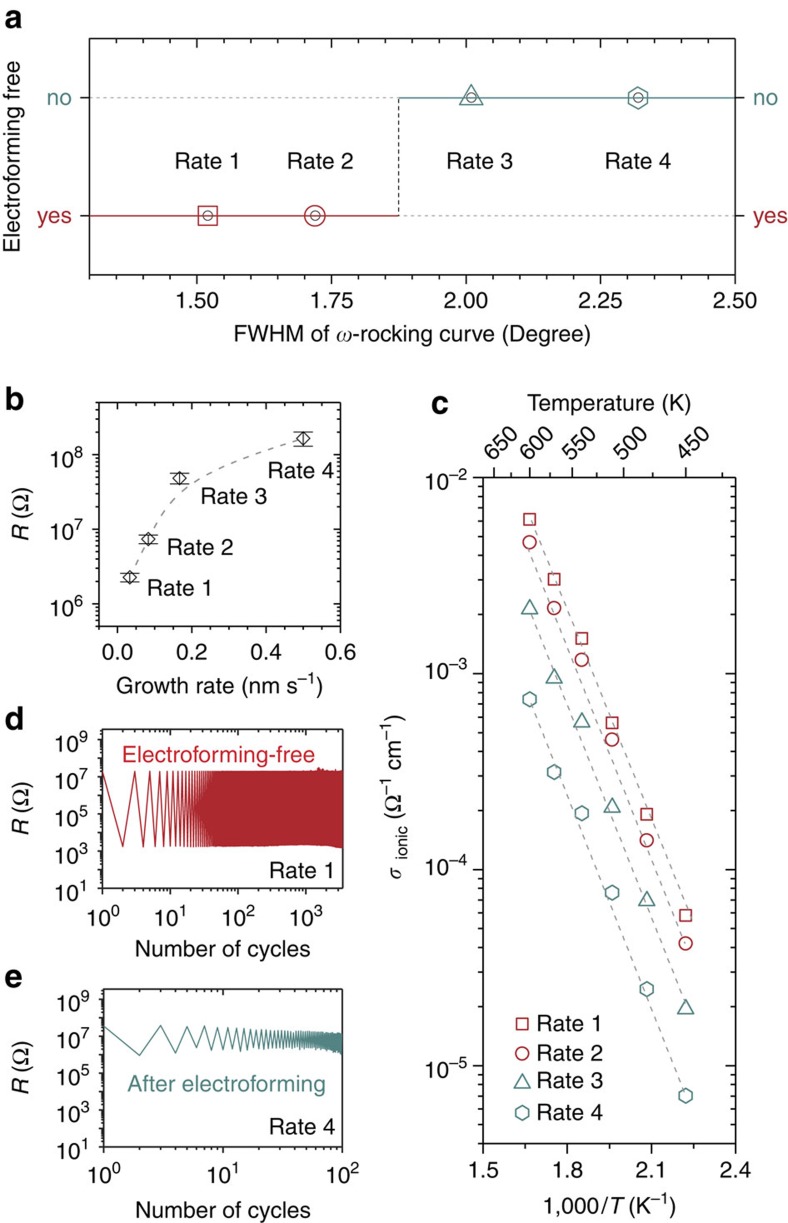
Electronic and ionic transport properties of VHN deposited from different film growth rates. (**a**) Electroforming-free resistive switching properties of SDC:STO VHN films depending on full width at half maximum (FWHM) values in *ω*-rocking curves; Rate 1 (red square); Rate 2 (red circle); Rate 3 (turquoise triangle); Rate 4 (turquoise hexagon). (**b**) Resistance values of the pristine states of the device with SDC:STO VHN films deposited from different growth rates (read voltage: 0.2 V). Error bars are provided as s.d. (**c**) Temperature-dependence of ionic conductivity (*σ*_ionic_) for SDC:STO VHN films deposited from different growth rates. (**d**,**e**) Resistance variation with repeated electrical cycles (with applied voltages of –5 and +5 V for switching and a read voltage of –0.3 V) of SDC:STO VHN film devices with different growth rates.

High-resolution TEM images (Supplementary Fig. 12) showed a less perfect lattice structure than the lower growth rate cases. Figure 6f shows XRD *ω*-rocking curves of SDC(002) peaks of the VHN films deposited at different growth rates. Higher growth rates led to higher full width at half maximum values in the *ω*-rocking curves indicating more tilted ionic and electronic channels and their degradation of the crystallinity, consistent with the TEM results. Figure 7a shows that electroforming-free resistive switching behaviour is only observed for the slowest grown samples which are the most aligned, highly crystalline samples with high ionic conductivity (high concentration of mobile oxygen vacancies). Indeed, Fig. 7b,c show the resistance values of pristine states at room temperature and the ionic conductivity (obtained from impedance spectroscopy measurements) for the different growth rates, respectively, showing clearly the samples grown at lower rates have higher electronic and ionic conductivity (a higher concentration of mobile oxygen vacancies). In the case of the two fastest grown samples, even after the electroforming process, low on–off ratios and poor endurance were exhibited, as shown in Fig. 7d,e. Hence, the results clearly show that high performance memristive devices are achieved only when films have a high concentration of mobile oxygen vacancies in the ionic SDC channels and when the ionic (SDC) and electronic channels (vertical interfaces) are well crystallized, highly aligned and connected through the film thickness.

## Discussion

A novel solid state ionic memristive composite oxide thin-film system was designed and demonstrated. These films, composed of separate ionic and electronic nanochannels gave electroforming-free resistive switching devices with highly tunable LRS and high HRS/LRS resistance ratios (∼10^4^). High switching variability between different devices combined with small HRS/LRS resistance ratios as shown by the majority of oxide ReRAMs, usually ∼10 to 10^2^ (ref. 38), have recently called the applicability of conventional oxide ReRAMs for memory arrays into question^39^. Forming-free ReRAMs with low variability and high on/off ratios, as shown by our devices, are therefore ideal model-systems for resistive switches.

The LRS was shown to correlate directly with the mobile oxygen vacancy concentration (ionic conductivity) in the SDC and was precisely tuned by varying the Sm doping level in the SDC. This tuning enabled high on–off ratios to be achieved. The device performance was improved significantly compared to the plain SDC film- and STO film-based devices which required high-voltage forming processes and have problems of reproducibility and endurance. Integration of memory devices in dense memory arrays is challenging for conventional oxide ReRAMs which rely on electroforming processes. After device fabrication, each device needs to be formed separately in a controlled manner due to the large deviation of the forming voltages usually observed. Moreover, the device parameters of access transistors (such as channel length/width and gate oxide thickness) to select individual memory cells need to be matched to the high forming voltages. Therefore, the design of forming-free oxide ReRAMs is also highly desirable from a practical point of view.

The form of *I*–*V* characteristics could be explained very clearly by invoking oxygen vacancy migration effects. The straightness and perfection of the ionic and electronic nanochannels was shown to be critical to the device performance. Nanocomposite-based microscale devices were demonstrated. To test the feasibility of nanoscale devices, we measured *I*–*V* cycles using a c-AFM tip on a vertical interface of the SDC:STO VHN film on the Nb:STO substrate (Supplementary Fig. 13). Reversible resistive switching behaviour was observed, which indicates that the VHN-based devices work with nanoscale electrodes.

We note that the physical cogency of a common ionic and electronic channel as presumed in the original model published by Strukov *et al*.^40^ and a violation of the Landauer principle, among others, have recently led to scientific dispute^41^,^42^. With the spatially separate ionic and electronic nanochannels found in our SDC:STO devices, we have experimentally proven for the first time that resistive switches can indeed be modelled by separate ionic and electronic channels^43^.

We also note that the high density (∼10^12^ inch^−2^) VHN structures in this work avoid degradation problems of dense, nanoscale features, which will be required for high density memories. Such nanoscale features formed from plain oxide films will require ion or electron beam milling steps which are problematic since these processes lead to surface oxygen defects and associated uncontrolled electronic conduction^44^.

There are however limitations of the nanocomposite model system studied in this work for practical ReRAM applications. First, the pulsed laser deposition process we use here is not in line/fully compatible with current integrated semiconductor manufacturing. In this context, epitaxial oxides can nowadays be grown by scalable, industrial growth routes such as reactive co-evaporation^45^. The nanocomposite films of this study could be grown in a similar way. Also, our devices have the potential to be integrated into state-of-the-art silicon technology since growth of epitaxial oxides and nanocomposite oxides on silicon is now well-established^46^,^47^. Second, relatively high voltages and currents were used in this experimental set-up for resistive switching. Thus, considerable power consumption is required for device operation. Third, the endurance we achieve is somewhat exceeding 10^3^ cycles, which is below the record 10^12^ cycles reported before^48^. On the other hand, the electroforming-free switching behaviour of our devices with better control over the built-in nanochannels promises space for improved device performance. For example, the use of integrated transistors close to the switching device to control the current in the LRS (ref. 49) is one way to achieve higher endurance and variability. Further improvement strategies should include doping of each nanocomposite component, and tuning of the nanocomposite materials and electrodes allowing separate ionic and electronic channel formation. In addition, the nanocolumn widths, which are controlled by the film growth rates, need to be larger than 17 nm for the devices to be electroforming-free (Fig. 7a, Rates 1 and 2). Concerning the scalability, switching on the 20 nm scale using c-AFM on a nanocolumn is feasible, suggesting that VHN-based devices work with nanoscale electrodes (Supplementary Fig. 13). However, the reported nanocolumn geometry can potentially result in large device to device variance (due to electrodes partially covering a nanocolumn). Therefore, enhancement of the nanocomposite film crystallinity is a key-aspect for applicability. By increasing the degree of crystallinity, electroforming-free properties can be achieved using a smaller nanocolumn width to potentially further reduce the device scale without producing a large device to device variance.

Finally, the use of the separate ionic and electronic nanochannel structures demonstrated here could have many benefits in other ionotronics areas, for example:

The spatial separation between conductance channels for different species is in good agreement with the classical Hodgkin–Huxley–Model^50^ for the simulation of biological neurons (for example, physically separate potassium and sodium ion channels). Here the two spatially separated channels, namely the ionic and electronic nanochannels, respectively, emulate spatially separated channels of bio-neurons. Such an analogy is not found in conventional memristive systems (where the ionic and electronic path are locally indistinguishable) making the spatially separate vertical channel structure a model system for artificial cognitive systems based on solid state ionotronics.

For ionic gating for transistors or memory applications, plain solid ionic films do not transfer ions sufficiently rapidly but the structures studied here have orders of magnitude higher ionic conduction^18^. Furthermore, there are several potential advantages of solid ionic memory devices over liquid ionically gated oxide memory devices which are being widely researched^51^,^52^,^53^,^54^.

For cathodes in fuel cells, mixed conductors are required but it is very difficult to have sufficient ionic and electronic conduction below 500 °C. The separate channel structures have overcome these limitations by inducing enhancements in both channels, controlled by careful materials selection. In addition, one can precisely tune the ion conductivity by adjusting the growth rate (Fig. 7c).

## Methods

### Film fabrication

Nanocomposite film growth was developed on the basis of that of 18. Films were grown on 0.5 wt.% Nb-doped SrTiO_3_ (001) single-crystalline substrates by pulsed laser deposition with a KrF laser (*λ*=248 nm) with a fluence of 1.5–4.5 J cm^−2^ and a repetition rate of 1–10 Hz. A polycrystalline 20 at.% SDC target, a polycrystalline STO target and polycrystalline targets containing SDC (Sm^3+^ concentrations: 0, 10, 20 and 30 at.%) and STO of 50:50 molar ratio were used for plain SDC films, plain STO films and SDC:STO VHN films, respectively. During deposition, the substrate temperature was 825 °C. Deposition was kept in an O_2_ atmosphere of 0.2 mbar. The samples were post-annealed at 700 °C for 1 h under 900 mbar O_2_. We deposited circular Pt electrodes by DC-magnetron sputtering onto the film surface with shadow masks for electrical measurements.

### Characterization

The phase and the crystalline quality of thin films were investigated by *ω*-2*θ* and asymmetric XRD on a PANalytical Empyrean high-resolution X-ray diffractometer using Cu-Kα radiation (*λ*=1.5405 Å). *ω*-rocking curves were obtained by measuring diffracted beam intensities around the SDC (002) as a function of the angle between incident X-rays and sample surface (*ω*). For investigating in-plane orientation, *ϕ* scans were obtained by 360° in-plane sample rotation around (111) peaks of the films and substrates. RSMs were collected about the STO (-203). *ω*-2*θ* diffraction peaks and RSM peaks were used to calculate lattice parameters of the films. An FEI TitanTM G2 80–200 STEM with a Cs probe corrector, operated at 200 kV was used to evaluate the structural properties across the interface. Cross-sectional samples were prepared by a standard manual grinding and thinning of samples with a final ion-milling step (Gatan PIPS 691 precision ion polishing system). The plan-view TEM sample was prepared by using focused ion beam with 3 keV Ga ions used as a final finishing step. The cross-sectional, and plan-view STEM images are taken in along [100] and [001] STO substrate direction, respectively. To determine film surface morphology, AFM (Multimode 8 SPM with Nanoscope V controller) was performed. To investigate local conduction at vertical interfaces, we employed an Agilent 5500 scanning probe microscope. Commercial silicon tips coated with platinum/iridium were used for conductive AFM. We measured the resistances using a two-probe station with a hot plate and a Keithley 2440 source-meter. To measure ionic transport characteristics with temperature variation, we used a hot plate and an HP 4294A Precision Impedance Analyser. For all measurements, Nb-doped STO substrates were grounded and the voltage is applied to the Pt electrodes.

### Data availability

The authors declare that the data supporting the findings of this study are available within the article and its Supplementary Information file. The numerical values of the data shown as the graphs are available upon request from the corresponding author.

## Additional information

**How to cite this article:** Cho, S. *et al*. Self-assembled oxide films with tailored nanoscale ionic and electronic channels for controlled resistive switching. *Nat. Commun.* 7:12373 doi: 10.1038/ncomms12373 (2016).

## Supplementary Material

Supplementary Information Supplementary Figures 1-13
