# Supplementary material for: Self-assembled oxide films with tailored nanoscale ionic and electronic channels for controlled resistive switching
Source: Nat Commun. 2016 Aug 5;7:12373. doi: 10.1038/ncomms12373 (PMC4980456; doi:10.1038/ncomms12373)
Supplement: Supplementary Information — Supplementary Figures 1-13 [file ncomms12373-s1.pdf]

Supplementary Information

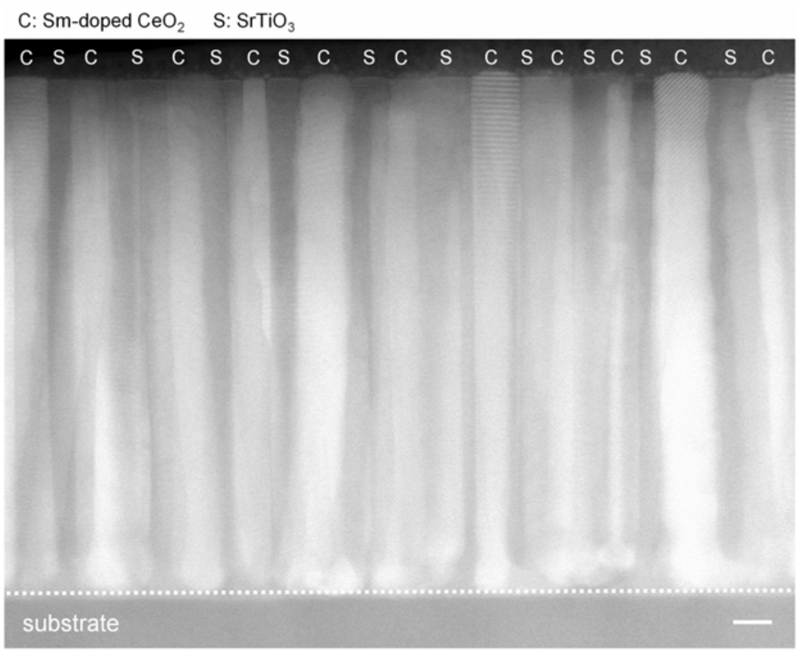

Supplementary Figure 1 | Scanning transmission electron microscopy (STEM). Cross-sectional STEM high-angle annular dark-field (HAADF) image of 20 at.% Sm-doped CeO_2:$SrTiO_3$ vertical heteroepitaxial nanocomposite film. Scale bar, 20 nm.

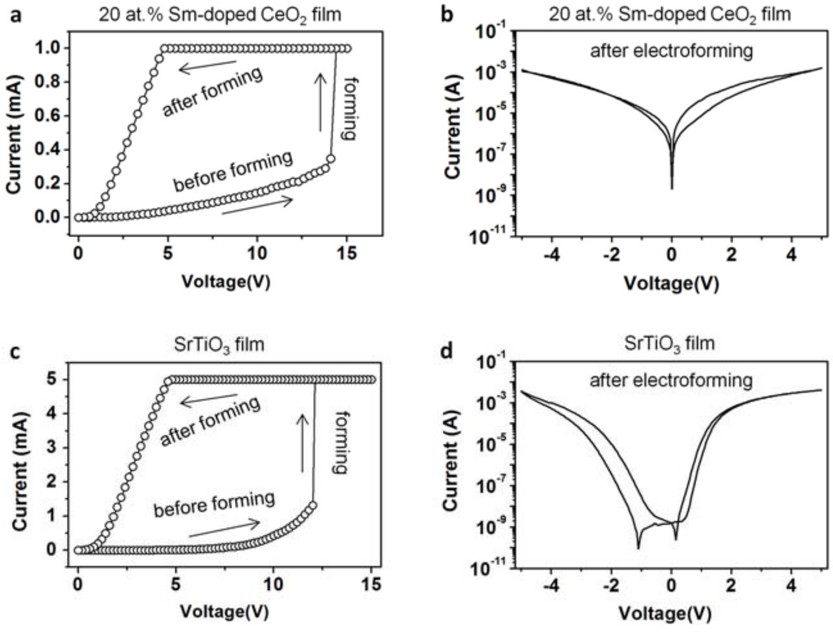

Supplementary Figure 2 | *I-V* characteristics of plain film devices. (**a** and **b**) 20 at.% Sm-doped CeO_2 film-based device. (**c** and **d**) $SrTiO_3$ film-based device.

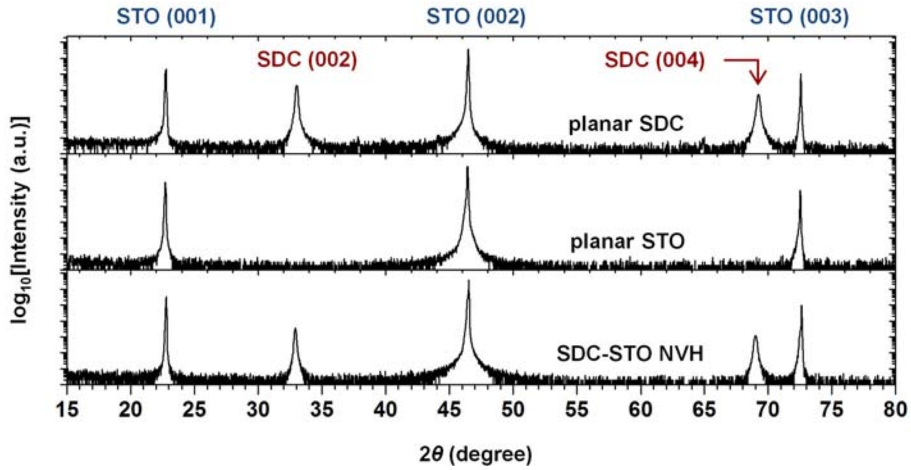

Supplementary Figure 3 | X-ray diffraction (XRD) measurements. XRD ω-2θ scans of plain 20 at.% Sm-doped CeO_2 (SDC) film, plain $SrTiO_3$ (STO) and SDC:STO vertical heteroepitaxial nanocomposite (VHN) film on 0.5 wt.% Nb-doped STO substrates.

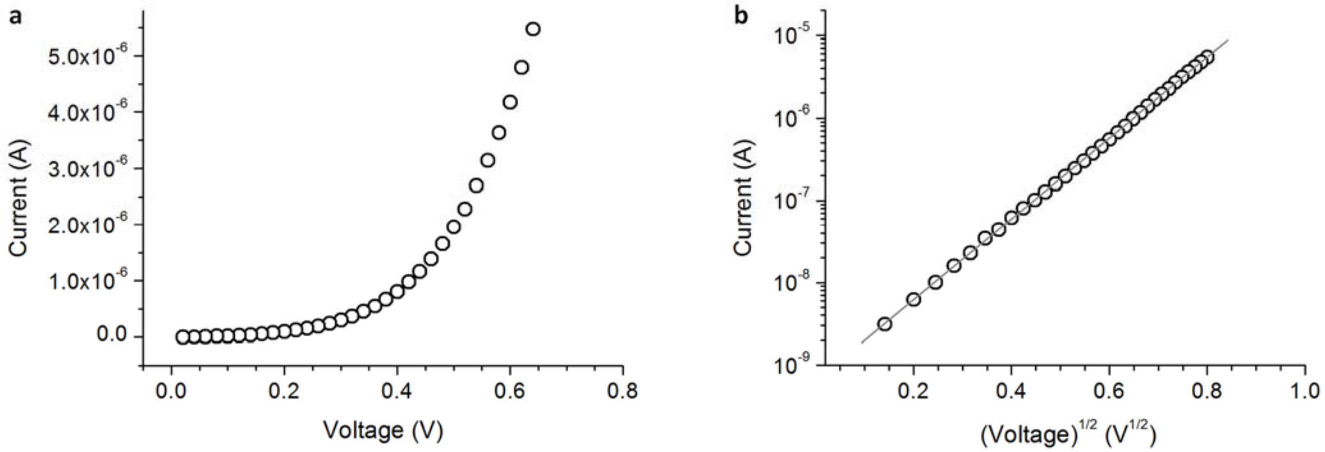

Supplementary Figure 4 | *I*-*V* characteristics of 20 at.% Sm-doped CeO_2:$SrTiO_3$ vertical heteroepitaxial nanocomposite device in high-resistance state. (**a**) *I*-*V* plot. (**b**) Semi-log I-$V^{1/2}$ plot.

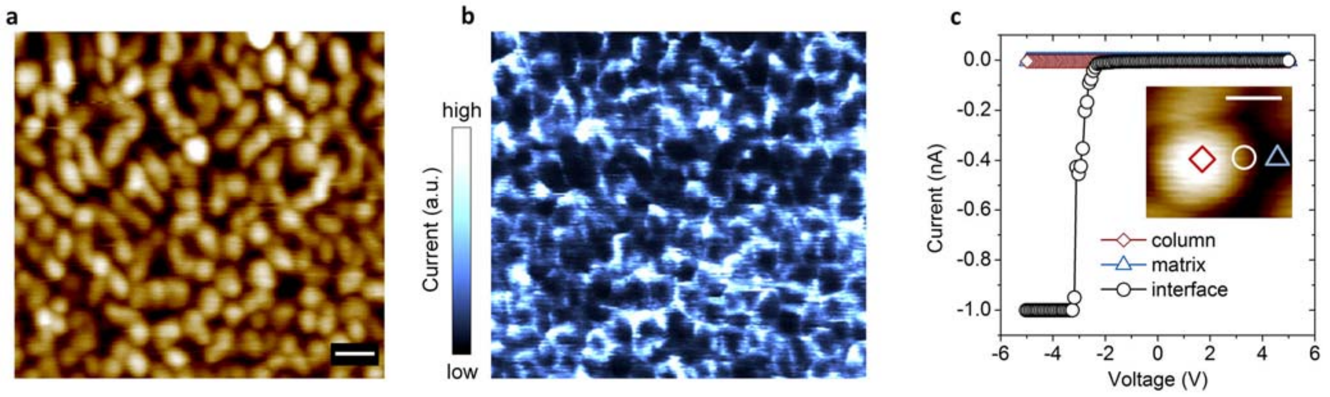

Supplementary Figure 5 | Atomic force microscopy. (**a**) Topography and (**b**) a corresponding current map of 20 at.% Sm-doped CeO_2:$SrTiO_3$ vertical heteroepitaxial nanocomposite film. Scale bar, 50 nm. (**c**) *I*-*V* curves at nanocolumn, interface and matrix. Scale bar, 20 nm.

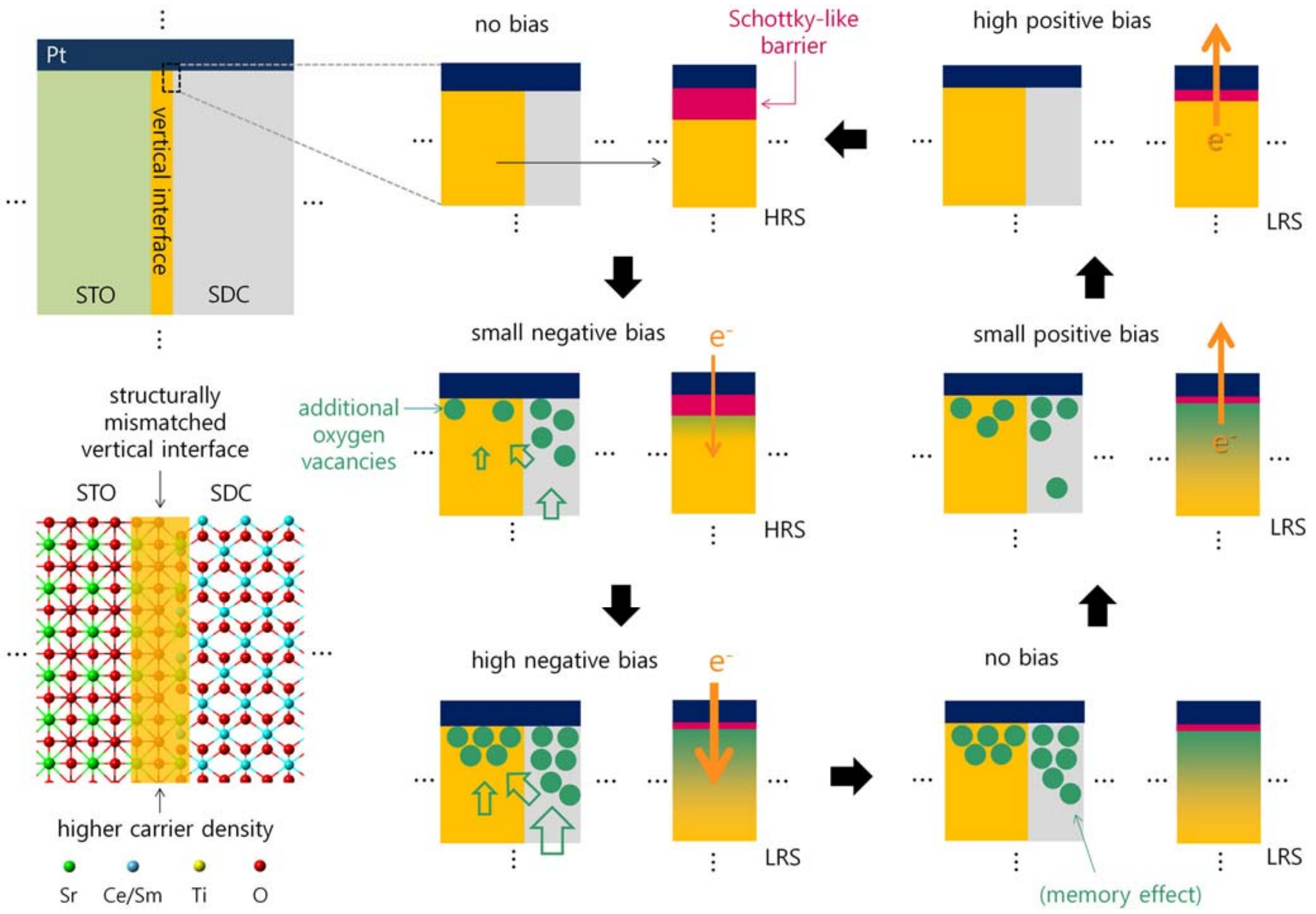

Supplementary Figure 6 | Schematic for interface between vertical interfaces and top electrode. Schematic showing the vertical interface formed between STO and SDC in the nanocomposite films, explaining the re-distribution of oxygen vacancies upon application of different biases, leading to Schottky-like barrier modulation at the top electrode interface with the vertical interfaces in the film.

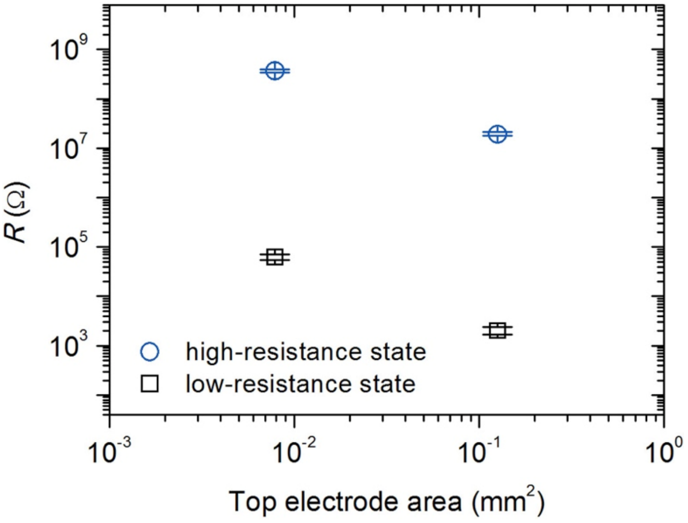

Supplementary Figure 7 | Device area-dependent resistance values. Resistance values in high-resistance states and low-resistance states of 20 at.% Sm-doped CeO_2:$SrTiO_3$ vertical heteroepitaxial nanocomposite devices with different Pt electrode areas. Error bars are provided as standard deviation.

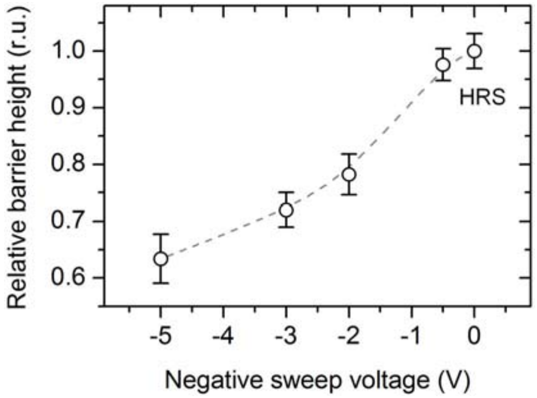

Supplementary Figure 8 | Emission barrier modulation. Relative emission barrier heights as a function of negative sweep voltages from the temperature-dependent conduction measurement (293 K, 313 K, 333 K and 353 K). Error bars are provided as standard deviation.

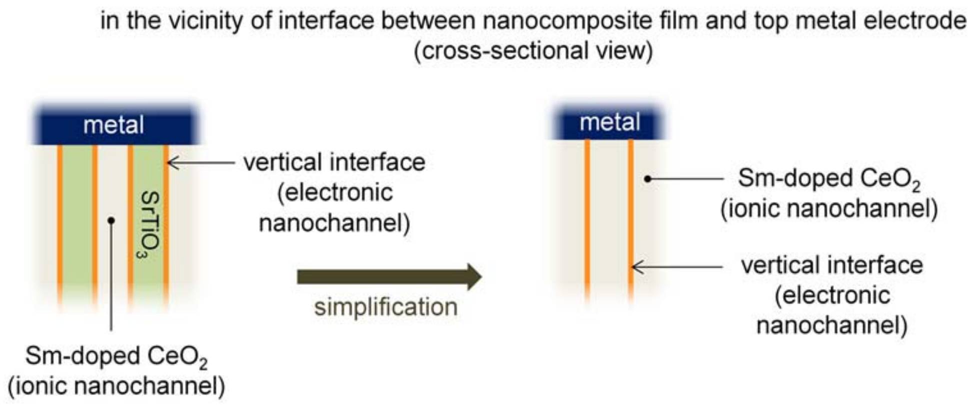

Supplementary Figure 9 | Simplification of schematic showing cross-sectional nanocomposite film device. Simplification of nanocomposite structure which is composed of 3 components of an ionically conducting Sm-doped CeO_2 (SDC) channel, a non-conducting 'bulk' $SrTiO_3$ (STO) channel, and an electronically conducting vertical interface channel (left image). This is done by discarding the 'bulk' STO part to show only the active regions of the SDC channel and the interface channel (right image). The simplified image is used in Figure 3.

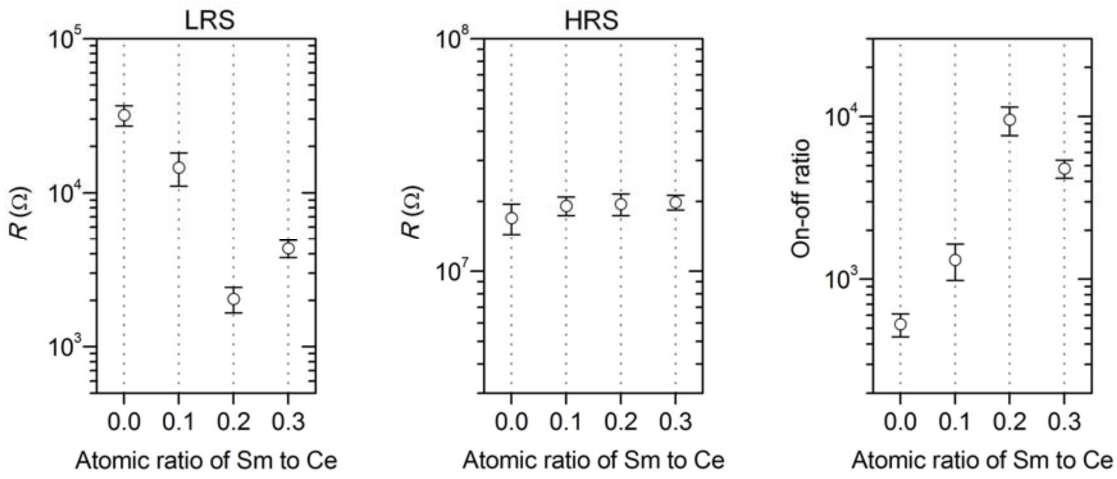

Supplementary Figure 10 | Dopant concentration-dependent resistance values. Resistance values in the low-resistance state (LRS) and high-resistance state (HRS) and on-off ratios as a function of dopant concentrations. Error bars are provided as standard deviation.

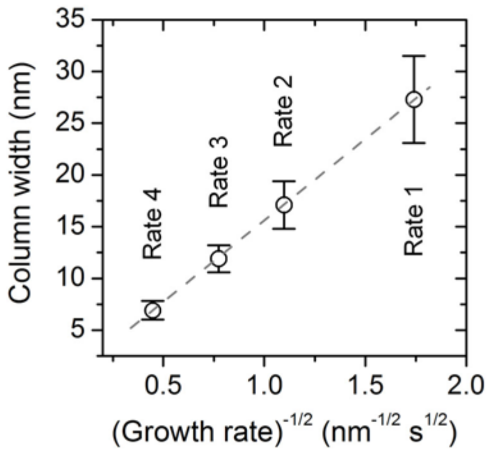

Supplementary Figure 11 | Diffusion length equation fitting. Column widths of 20 at.% Sm-doped CeO_2:$SrTiO_3$ vertical heteroepitaxial nanocomposite films as a function of (growth rate)$^{-1/2}$. Error bars are provided as standard deviation.

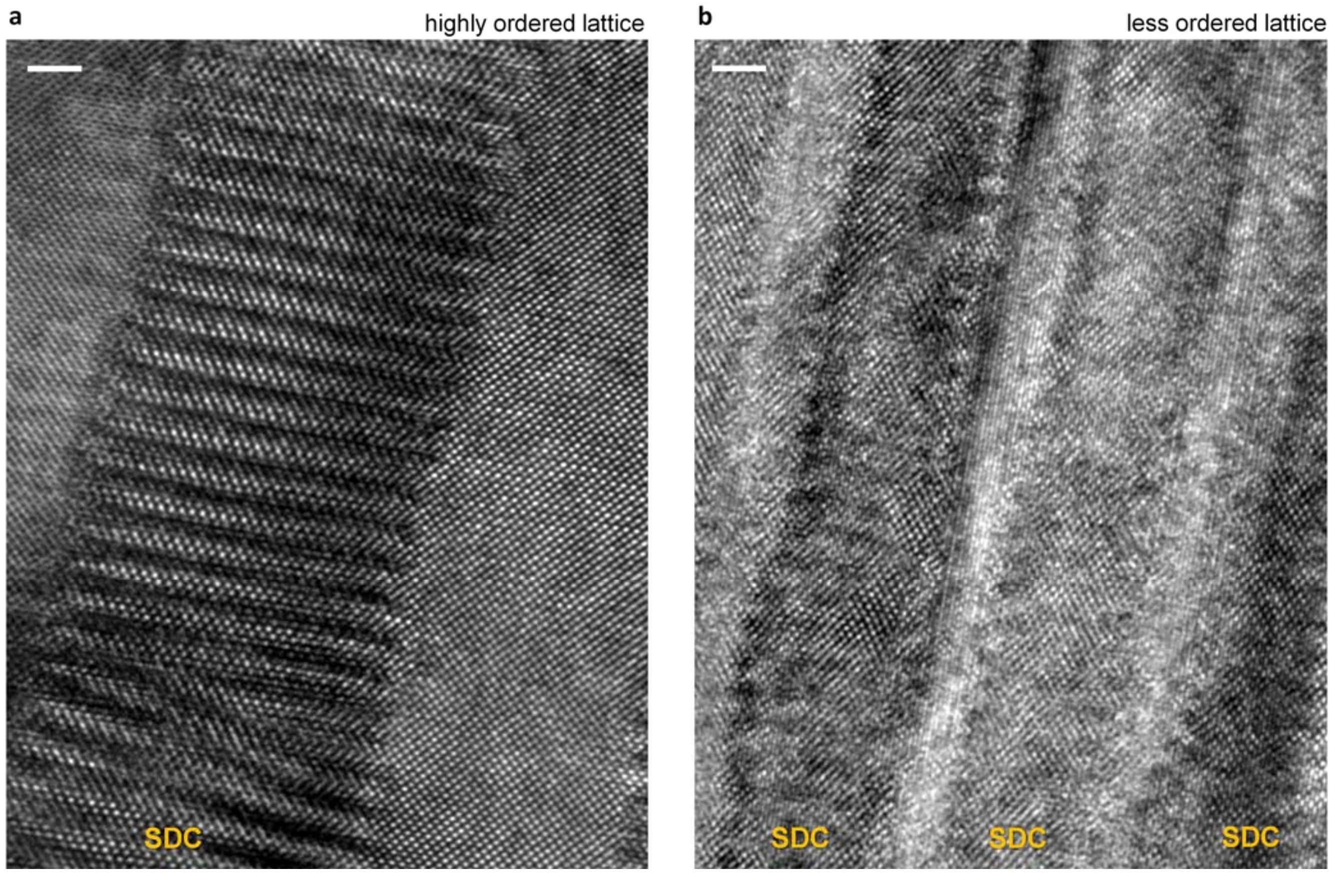

Supplementary Figure 12 | High-resolution transmission electron microscopy (HR-TEM) on films deposited from different growth rates. Cross-sectional HR-TEM image of 20 at.% Sm-doped CeO_2:$SrTiO_3$ vertical heteroepitaxial nanocomposite films deposited from different growth rates: (**a**) Rate 2 (0.08 nm s^{-1}). (**b**) Rate 4 (0.50 nm s^{-1}). Scale bars, 2 nm.

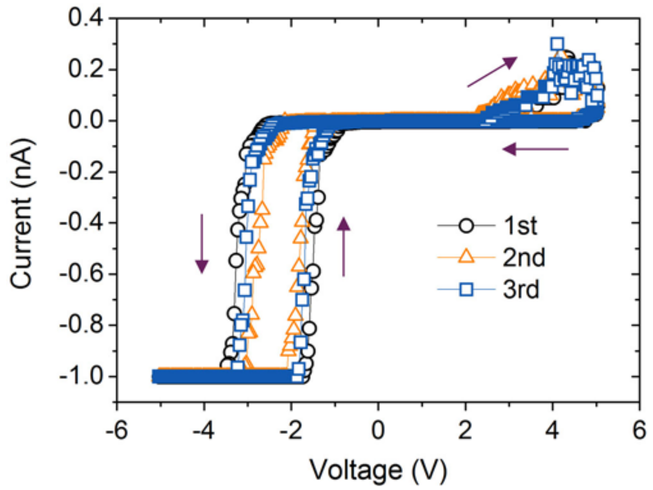

Supplementary Figure 13 | Feasibility test for nanoscale device. *I*-*V* cycles measured using conductive atomic force microscope on a vertical interface of a 20 at.% Sm-doped CeO_2:$SrTiO_3$ (STO) vertical heteroepitaxial nanocomposite film grown on a Nb-doped STO substrate.
